# ECMO Rescues Patients With Acute Respiratory Failure Related to GPA

**DOI:** 10.3389/fmed.2021.671396

**Published:** 2021-05-28

**Authors:** Rongjun Wan, Wenzhe Yang, Xinhua Ma, Wei Yang, Pinhua Pan, Chengping Hu, Qiong Chen, Yaou Zhou, Rongli Lu, Yimin Fang, Yuanyuan Li

**Affiliations:** ^1^Department of Respiratory Medicine (Department of Respiratory and Critical Care Medicine), Xiangya Hospital, Central South University, Changsha, China; ^2^National Clinical Research Center for Geriatric Disorders, Xiangya Hospital, Central South University, Changsha, China; ^3^Department of Critical Care Medicine, Xiangya Hospital, Central South University, Changsha, China; ^4^Department of Rheumatology and Immunology, Xiangya Hospital, Central South University, Changsha, China

**Keywords:** diffuse alveolar hemorrhage, ANCA-associated vasculitis, extracorporeal membrane oxygenation, acute respiratory failure, granulomatosis with polyangiitis

## Abstract

Granulomatosis with polyangiitis (GPA) is a subtype of anti-neutrophil cytoplasmic antibody-associated vasculitis with a wide range of clinical symptoms related to the systemic involvement of small blood vessels. The respiratory system is one of the most frequently involved, and life-threatening acute respiratory failure could occur due to diffusive alveolar hemorrhage and tracheal stenosis. When maximum mechanical ventilation is unable to maintain oxygenation, extracorporeal membrane oxygenation (ECMO) should be considered as the final respiratory supportive method, if available. Here we present a 32-year-old male patient with acute respiratory failure (ARF) related to GPA, who was rescued by winning time for accurate diagnosis and appropriate treatment. Additionally, we reviewed more than 60 GPA-related ARF cases on multiple online databases, summarized the clinical manifestations of these patients, and concluded that ECMO plays an important role in further respiratory support for ARF patients with GPA and assists in accurate and timely diagnosis and appropriate treatment, thus helping them recuperate.

## Introduction

Granulomatosis with polyangiitis (GPA), formerly termed Wegener's granulomatosis, is the most common pathogenesis of anti-neutrophil cytoplasmic antibody (ANCA)-associated vasculitis (AAV) and highly relate to PR3-ANCA (1). The diversiform characteristics of GPA can refer to almost all body systems, particularly the lungs and kidneys (2). Pulmonary hemorrhagic nephritis syndrome is the most frequent cause of acute respiratory failure (ARF), and intensive care unit (ICU) supervision with advanced respiratory support and renal replacement treatment is necessary to help those patients recuperate (3, 4).

Extracorporeal membrane oxygenation (ECMO) is the final respiratory support technique for severe acute respiratory distress syndrome (ARDS) after maximum mechanical ventilation fails to provide optimal oxygen levels in the blood (5, 6). Although systemic anticoagulation is required during ECMO, with the advancement of technology, diffuse alveolar hemorrhage (DAH) has become a potential indication of ECMO as well.

**Figure 1:**
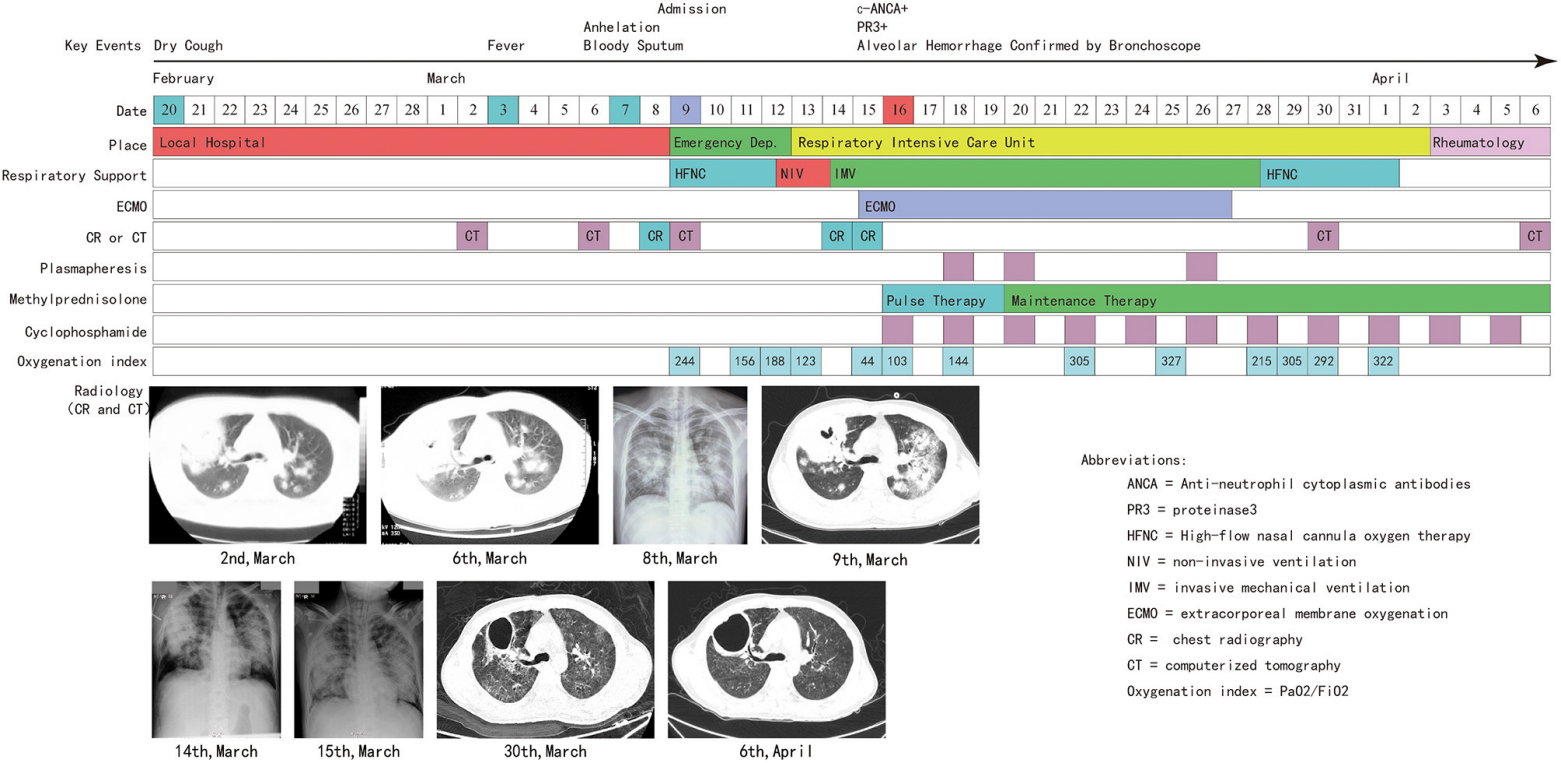
Time-line of courses and treatments.

According to a literature review, only the respiratory system is involved in patients with GPA, and ARF at the onset of symptoms is rare (7). Here we report a case of GPA with ARF at the onset of the disease. The disease progressed from shortness of breath to severe respiratory failure in 3 weeks, and maximum mechanical ventilation could not maintain oxygenation. The patient eventually recovered after venovenous extracorporeal membrane oxygenation (VV-ECMO), plasmapheresis, and immunosuppressive treatment (Figure 1). Within 2 years of follow-up, the patient showed a great prognosis.

In this study, we also reviewed 60 cases of ARF caused by GPA as published in academic journals; among this series, 22 patients recovered successfully after ECMO support.

## Case Report

On March 13, 2018, a 32-year-old male patient was admitted to the respiratory acute ICU (RICU) at our hospital owing to cough, expectoration, dyspnea for 3 weeks, and fever for 1 week. At 3 weeks before admission, he developed a dry cough and fever sequentially, and the fever was irregular with the highest temperature of 38.0°C, and it was difficult to get a reduction through centrifugation. In a local hospital, a computed tomography (CT) scan showed multiple shadows, and physicians prescribed demethylvancomycin, meropenem, and oseltamivir based on an empirical diagnosis of community-acquired pneumonia (CAP). However, the anti-microorganism treatment was ineffective, and the patient progressed into expectorating blood-stained sputum and dyspnea. For improved treatment, the patient was transferred to the emergency department on March 9. Before he became ill, he worked as a metal mechanical processing engineer for 8 years and had a history of metallic dust and chemical aerosol inhalation. He also had a smoking history of 1.25 pack-years.

After admission to our hospital, the patient reported aggravation of dyspnea with white mucus sputum. Physical examination showed that his body temperature was between 38.0 and 39.0°C without downtrend; the respiratory rate was 30 times/min, auscultation demonstrated only a reduction of breath sound at both sides of the lung, and no rales were revealed. There were no hemorrhagic spots or rashes on the skin. Laboratory examinations showed elevated procalcitonin, C-reactive protein, and erythrocyte sedimentation rate. On CT re-scan in this patient, multiple cavities emerged in his right lung. An empirical diagnosis of severe CAP was established. On estimation, methicillin-resistant *Staphylococcus aureus* or fungus was the most probable pathogen; thus, the patient was treated using linezolid, voriconazole, and levofloxacin. However, the patient continued to develop dyspnea and hyperpyrexia, indicating the probability of incorrect diagnosis. Simultaneously, the patient's blood gas analysis revealed a type I respiratory failure, with an oxygenation index of approximately 100. Although high-flow mask oxygen inhalation and non-invasive positive pressure ventilation (NIPPV) were administered sequentially, the blood oxygenation worsened, and the patient was transferred to our RICU on March 8. Considering that NIPPV did not improve oxygenation, a bedside chest film revealed consolidation in the lungs bilaterally, and the oxygenation index dropped to 60. Hence, bedside bronchoscope-guided intubation and invasive ventilation were performed. Although maximum ventilatory support was provided under sedation and analgesia, the oxygenation index did not show any improvement at 5 h later. Based on the small amount of hemorrhagic secretion in the trachea under bronchoscopy and consolidation of the gravity areas of the lower lungs by critical ultrasonic imaging, DAH was suspected, and more advanced life support systems were planned. Thus, our team performed VV-ECMO through the internal jugular and femoral veins. The ventilator was changed to synchronized intermittent mandatory ventilation sequentially, and blood gas analysis improved immediately (Table 1). Thus, the diagnosis of potential connective tissue diseases such as pulmonary vasculitis was made.

**Table 1:** Extracorporeal membrane oxygenation (ECMO) and SIMV parameters and results of arterial blood gas analysis.

**Time**	**ECMO**				**SIMV**					**Blood gas analysis**			
	**Pump (rpm)**	**Blood flow (L/min)**	**Air flow (L/min)**	**Air FiO_**2**_ (%)**	**RR (/min)**	**TV (ml)**	**Pressure (cmH_**2**_O)**	**PEEP (cmH_**2**_O)**	**Air FiO_**2**_ (%)**	**pH**	**pCO_**2**_**	**pO_**2**_**	**OI(pO_**2**_/FiO_**2**_)**
1st day (before ECMO)	-	–	–	–	44–54 (SPONT)	–	12	8–20	100	7.29	59	44	44
1st day (after ECMO)	3,485	3.8	5	100	14	380	10	12	60	7.43	36	65	65
2nd day	3,510	4.34	7	100	14	240	10	12	50	7.45	41	62	103
3rd day	3,555	4.33	6	100	–	–	–	–	–	–	–	–	–
4th day	–	–	–	–	14	223	10	12	50	7.41	48	72	144
6th day	3,555	4.32	8	100	–	–	–	–	50	–	–	–	–
7th day	3,555	4.23	7.5	100	–	–	–	–	50	–	–	–	–
8th day	3,500	4.02	8	100	–	–	–	–	50	7.49	43	120	305
9th day	–	–	–	–	14	244	10	10	40	–	–	–	–
10th day	3,480	4	8	100	–	–	–	–	40	–	–	–	–
11th day	3,480	3.64	8	100	14	240	10	10	40	7.46	44	131	327
12th day (removal of ECMO)	3,480	4	6.5	40	14	380	10	14	60	7.51	40	129	215
13th day (HFNC)	–	–	–	–	–	–	–	–	–	7.47	38	146	292
15th day	–	–	–	–	–	–	–	–	–	7.48	38	129	322

*SIMV, synchronized intermittent mandatory ventilation; RR, respiratory rate; TV, tide volume; OI, oxygenation index; PEEP, positive end–expiratory pressure; SPONT, continuous spontaneous ventilation; HFNC, high–flow nasal cannula oxygen therapy; –, not applicable or no data or not changed*.

On the day after ECMO was established, the patient's immune disorder-related test results were back, with negative anti-nucleic antibody and anti-dsDNA antibody and positive cytoplasmic anti-neutrophil cytoplasmic antibody (c-ANCA) and proteinase3 (PR3) antibodies; we confirmed the patient's diagnosis as GPA. Considering medical images, airway hemorrhagic secretion, and results of positive autoantibodies, the ARF of this patient was suspected to be due to DAH. Immunosuppressive therapy immediately replaced the antibiotics. Methylprednisolone impact therapy (March 16–18, 500 mg Qd) and maintenance therapy (March 20 and later, 40 mg Qd), cyclophosphamide (0.2 g, Qod), and plasmapheresis (March 19, 21, and 27; three times in total) were administered. Subsequently, the oxygenation and lung conditions of this patient gradually improved (Figure 1, Table 1). On the 12th day after ECMO, the latter was removed after interrupting the oxygen source of ECMO for 4 h. At 1 day later, intubation was removed after evaluation. High-flow nasal cannula oxygen therapy (HFNC) was observed to be sufficient to maintain oxygenation. On April 3, the patient was transferred to the rheumatology department and treated for another 4 days. On April 6, the patient was discharged in a good respiratory condition.

**Figure 2:**
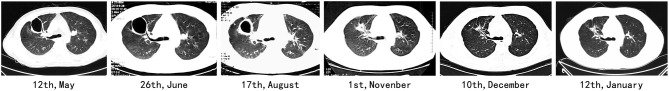
Chest CT scans at follow up.

During follow-up, methylprednisolone and cyclophosphamide were maintained, and the dosages were gradually reduced, and the patient showed a stable condition. Multiple CT scans showed diminishing cavities; however, fibrosis persisted (Figure 2).

## Methods

### Data Collection

A literature search was performed in MEDLINE/PUBMED, Embase, and Scopus databases using the terms, “respiratory failure,” “Wegener's granulomatosis,” and “granulomatosis with polyangiitis.” The article type was limited to case reports when eligible literature was collected from Embase. Furthermore, wild retrieval was performed through Google Scholar. All conference papers and reports that were not in English were excluded, owing to a lack of medical details. By reading the abstract and full text, we collected data on patients with respiratory failure related to GPA, and the inclusion criteria were as follows: (1) diagnosed with GPA, (2) acute respiratory failure and mechanical ventilation, and (3) the patient's medical history was described in detail. We reviewed the basic information, clinical characteristics, laboratory results, radiology characteristics, ECMO application and type, treatment, and prognosis among these patients.

### Statistical Analysis

We used the Wilcoxon rank-sum test to assess the statistical difference in the age between patients who were administered ECMO or not. We also used Fisher's exact test to examine differences of other clinical characteristics between the two groups. Patients who lacked specific items were excluded when performing statistical analyses on relative items. *P* < 0.05 was considered significant. All the analyses were performed in R software (version 3.6.3).

## Results

The overall schematic general information and clinical manifestations are presented in Table 2. Among the 61 cases in this review (including our case), 32 were women (32/61). The mean age was 35.0 years (interquartile range, IQR: 32.0–57.0). However, the mean age of patients who received ECMO was 27.0 years (IQR, 20.8–33.8), those who did not receive ECMO was 51.0, and their IQR was 30.0–61.5, much higher than that of patients who received ECMO treatment (Table 3).

**Table 2:** Case reports of respiratory failure due to granulomatosis with polyangiitis.

**Case**	**Author**	**Publication year**	**Age**	**Gender**	**Respiratory onset**	**Extrapulmonary involvement**	**Reason of respiratory failure**	**ANCA**		**Radiology**	**ECMO**		**Immune-suppression**	**Plasmapheresis**	**Rituximab**	**Outcome**
								**Type**	**Antigen**		**Use and type**	**Duration (days)**				
1	Leak et al. (8)	1967	61	Female	No	Yes	DAH	–	–	Infiltration, nodule	Not used	–	GC	No	No	Death
2	Leak et al. (8)	1967	45	Female	Yes	Yes	DAH	–	–	Cavity, infiltration	Not used	–	GC	No	No	Discharged
3	Hensley et al. (9)	1979	57	Female	No	Yes	DAH	–	–	Infiltration	Not used	–	GC, Cyc	No	No	Discharged
4	Feinstein et al. (10)	1985	22	Male	No	Yes	DAH	–	–	Cavity, infiltration	Not used	–	GC, Cyc	Yes	No	Discharged
5	Grupe and Colvin (11)	1986	15	Male	No	Yes	DAH	–	–	Infiltration	Not used	–	GC, Cyc	Yes	No	Death
6	Lenclud et al. (12)	1989	69	Female	Yes	Yes	DAH	p	–	Infiltration	Not used	–	GC, Cyc	No	No	Death
7	Lenclud et al. (12)	1989	70	Male	Yes	Yes	DAH	Positive	–	Infiltration	Not used	–	GC, Cyc	No	No	Discharged
8	Coggins et al. (13)	1989	56	Male	Yes	Yes	DAH	p/c	PR3/MPO	Infiltration	Not used	–	GC, Cyc	Yes	No	Death
9	Sanchez et al. (14)	1989	82	Female	Yes	Yes	DAH	–	–	Infiltration	Not used	–	GC, Cyc	No	No	Death
10	Misset et al. (15)	1991	35	Female	Yes	Yes	DAH	Negative	–	Infiltration	Not used	–	GC, Cyc	No	No	Discharged
11	Yoshimura et al. (16)	1992	58	Male	No	Yes	DAH	c	–	Nodule	Not used	–	GC, Cyc	No	No	Death
12	Odeh, M. et al. (17)	1993	75	Female	No	Yes	DAH	–	–	Consolidation	Not used	–	GC, Cyc	No	No	Death
13	Pradhan et al. (18)	2000	15	Female	Yes	Yes	DAH	p	MPO	Nodule	Not used	–	GC, Cyc	No	No	Discharged
14	Ullmer et al. (19)	2000	62	Male	Yes	Yes	DAH/PCP	c	–	Nodule, infiltration	Not used	–	GC, Cyc	No	No	Discharged
15	Hermon et al. (20)	2003	17	Female	No	Yes	DAH	c	PR3	Infiltration	Not used	–	GC, Cyc	No	No	Discharged
16	Senf et al. (21)	2003	35	Male	No	Yes	DAH/hydrothorax	c	PR3	Nodule, infiltration	Not used	–	GC, Cyc	Yes	Yes	Discharged
17	Prutkin et al. (22)	2003	56	Male	Yes	Yes	DAH	c	PR3	Infiltration, consolidation	Not used	–	GC, Cyc	No	No	Discharged
18	Griffith et al. (23)	2003	28	Male	Yes	Yes	DAH	c	PR3	Infiltration	Not used	–	GC, Cyc	Yes	No	Discharged
19	Steinau et al. (24)	2003	19	Male	Yes	Yes	DAH	c	PR3	Infiltration	Not used	–	GC, Cyc	No	No	Discharged
20	Nguyen et al. (25)	2005	20	Male	No	Yes	DAH	c	–	Infiltration	Not used	–	GC, Cyc	Yes	No	Discharged
21	Mera et al. (26)	2007	19	Male	Yes	Yes	DAH	c	PR3	Cavity, nodule	Not used	–	GC, Cyc	No	No	Discharged
22	Mukhopadhyay et al. (27)	2010	37	Male	Yes	Yes	DAH	c	PR3	Consolidation	Not used	–	(Death)	No	No	Death
23	Esposito et al. (28)	2010	7	Female	Yes	Yes	DAH	c	PR3	Infiltration, consolidation	Not used	–	GC, Cyc	No	No	Discharged
24	Berthoux et al. (29)	2011	54	Male	No	Yes	DAH	c	PR3	Consolidation	Not used	–	GC, Cyc	Yes	Yes	Discharged
25	Mahajan et al. (30)	2011	35	Male	Yes	Yes	DAH	c	–	Infiltration	Not used	–	GC, Cyc	No	No	Discharged
26	Marina et al. (31)	2011	64	Female	Yes	Yes	DAH/mucormycosis	c	PR3	Infiltration, nodule	Not used	–	GC, Cyc	Yes	No	Death
27	Cardenas–Garcia et al. (32)	2012	51	Female	Yes	Yes	DAH	c	–	Infiltration	Not used	–	GC, Cyc	Yes	No	Discharged
28	Ishiguro et al. (33)	2012	73	Female	No	Yes	DAH/CMV	Positive	PR3	Infiltration, nodule	Not used	–	GC, Cyc	No	No	Death
29	Kaya et al. (34)	2013	48	Male	No	Yes	DAH	c	PR3	Infiltration, nodule	Not used	–	GC, Cyc	Yes	No	Death
30	Powers et al. (35)	2013	32	Male	Yes	Yes	DAH	c	PR3	Infiltration, consolidation, nodule	Not used	–	GC, Cyc	Yes	Yes	Discharged
31	Haupt et al. (36)	2013	14	Female	Yes	Yes	DAH	c	PR3	Infiltration	Not used	–	GC	Yes	No	Discharged
32	Pinto et al. (37)	2014	65	Female	Yes	Yes	DAH/pneumothorax	c	PR3	Cavity	Not used	–	GC, Cyc	No	No	Death
33	Kaya et al. (34)	2014	60	Male	No	Yes	DAH	c	PR3	Infiltration	Not used	–	GC, Cyc	Yes	No	Discharged
34	Moreno-González et al. (38)	2014	42	Male	Yes	Yes	DAH/PTE	Positive	PR3	Infiltration, nodule	Not used	–	GC, Cyc	Yes	Yes	Discharged
35	Hilal et al. (39)	2015	55	Female	Yes	Yes	DAH/CMV	c	PR3	Infiltration	Not used	–	GC	Yes	Yes	Death
36	Fukui et al. (40)	2015	69	Male	No	Yes	DAH/CMV	c	MPO	Consolidation	Not used	–	GC, Cyc	Yes	No	Death
37	Tajarernmuang et al. (41)	2016	48	Female	No	Yes	Airway stenosis	c	PR3	Stenosis	Not used	–	GC, Cyc	No	No	Discharged
38	Ning et al. (42)	2018	70	Female	Yes	Yes	DAH	c	PR3	Nodule, infiltration, cavity	Not used	–	GC, MMF	Yes	No	Discharged
39	Sattar et al. (43)	2019	60	Female	No	Yes	DAH	c	PR3	Cavity, infiltration	Not used	–	GC, Cyc	Yes	No	Discharged
40	Hartmann et al. (44)	1994	20	Female	No	Yes	DAH	c	–	Infiltration	VV	6	GC, Cyc	Yes	No	Discharged
41	Loscar et al. (45)	1997	19	Female	No	Yes	DAH	c	PR3	–	VV	10	GC, Cyc	No	No	Discharged
42	Matsumoto et al. (46)	2000	29	Female	Yes	Yes	DAH	c	–	Nodule, infiltration	VA	3.4	GC, Cyc	No	No	Discharged
43	Rosengarten et al. (47)	2002	27	Female	Yes	Yes	DAH	c	PR3	Infiltration	VV	15	GC, Cyc	No	No	Discharged
44	Hernandez et al. (48)	2002	13	Male	Yes	Yes	DAH	c	–	Infiltration	VV	9	GC, Cyc	Yes	No	Discharged
45	Ahmed et al. (49)	2004	26	Female	Yes	Yes	DAH	c	–	Cavity, infiltration	VV	14	GC, Cyc	Yes	No	Discharged
46	Gay et al. (50)	2005	26	Female	Yes	Yes	DAH	c	–	Cavity, infiltration	VV	10	GC, Cyc	Yes	No	Discharged
47	Balasubramanian et al. (51)	2005	53	Male	No	Yes	DAH	c	PR3	Infiltration	VV	NA	GC, Cyc	Yes	No	Discharged
48	Joseph et al. (52)	2011	13	Male	Yes	Yes	DAH	Positive	PR3	Infiltration	VV	7	GC	Yes	No	Discharged
49	Barnes et al. (53)	2012	50	Female	Yes	Yes	DAH	c	PR3	Consolidation	VV	1.5	GC, Cyc	Yes	No	Discharged
50	Hohenforst et al. (54)	2013	65	Female	Yes	Yes	DAH	Positive	PR3	Infiltration	VV	10	GC, Cyc	Yes	No	Discharged
51	Yusuff et al. (55)	2015	23	Female	Yes	Yes	DAH	c	PR3	–	VV	13	GC, Cyc	No	No	Discharged
52	Yusuff et al. (55)	2015	27	Male	Yes	Yes	DAH	c	–	Infiltration	VV	21	GC, Cyc	Yes	Yes	Discharged
53	Rawal et al. (56)	2016	28	Male	Yes	Yes	DAH	Positive	PR3	Infiltration	VV	21	GC, Cyc	Yes	No	Discharged
54	Vanoli et al. (57)	2017	33	Male	Yes	Yes	DAH	c	PR3	Consolidation, infiltration	VV	NA	GC, Cyc	Yes	Yes	Discharged
55	Falk et al. (58)	2017	17	Female	Yes	No	Airway stenosis	c	PR3	Emphysema	VA and VV	21	GC, Cyc	Yes	Yes	Discharged
56	Delvino et al. (59)	2019	45	Male	Yes	Yes	DAH	c	PR3	Infiltration	VV	6	GC, Cyc	Yes	No	Discharged
57	Delvino et al. (59)	2019	45	Male	No	Yes	DAH	c	PR3	Nodule, consolidation	VV	14	GC, MTX	No	Yes	Discharged
58	Goel et al. (60)	2020	34	Female	Yes	Yes	DAH	c	PR3	Nodule, consolidation	VV	7	GC, Cyc	Yes	No	Discharged
59	Yin et al. (61)	2020	25	Female	Yes	Yes	DAH	Positive	–	Infiltration, consolidation	VV	52	GC, Cyc	No	No	Discharged
60	Yin et al. (61)	2020	18	Female	Yes	Yes	DAH	Positive	–	–	VV	123	GC	Yes	Yes	Lung transplant
61	This case	2020	32	Male	Yes	No	DAH	c	PR3	Cavity, infiltration, consolidation	VV	12	GC, Cyc	Yes	No	Discharged

*PTE, pulmonary thromboembolism*.

**Table 3:** Summary of cases of respiratory failure due to GPA.

	**Total (IQR/%)**	**ECMO (IQR/%)**	**Non-ECMO (IQR/%)**	***P*-value**
	**(*n* = 61)**	**(*n* = 22)**	**(*n* = 39)**	
Publication time				0.08
Before 2000	17 (27.87)	3 (13.64)	14 (35.90)	–
After 2000	44 (72.13)	19 (86.36)	25 (64.10)	–
Age	35.00 (23.00–57.00)	27.00 (20.75–33.75)	51.00 (30.00–61.50)	<0.01
Female	32 (52.45)	13 (52.63)	19 (53.13)	0.59
Respiratory onset	41 (67.21)	18 (81.82)	23 (58.97)	0.09
ANCA				0.80
No information	7 (11.48)	0	7 (17.95)	–
Negative	1 (1.64)	0	1 (2.56)	–
Unknown type	8 (13.11)	5 (22.72)	3 (7.69)	–
c-ANCA	42 (68.85)	17 (77.27)	25 (64.10)	–
p-ANCA	2 (3.28)	0	2 (5.13)	–
p + c-ANCA	1 (1.64)	0	1 (2.56)	–
ANCA antigen				0.69
No information	23 (37.70)	8 (36.36)	15 (38.46)	–
PR3	35 (57.38)	14 (63.64)	21 (53.85)	–
MPO	2 (3.28)	0	2 (5.13)	–
PR3 + MPO	1 (1.64)	0	1 (2.56)	–
**Radiology**				
No information	3 (4.92)	3 (13.64)	0	–
Infiltration	45 (73.77)	15 (68.18)	30 (76.92)	0.55
Consolidation	13 (21.31)	6 (27.27)	7 (17.95)	0.07
Cavity	9 (14.75)	3 (13.64)	6 (15.38)	1
Nodule	15 (24.59)	3 (13.64)	12 (30.77)	0.22
Stenosis	1 (1.64)	0	1 (2.56)	1
Emphysema	1 (1.64)	1 (4.55)	0	0.36
Treatment				
GC	60 (98.36)	22 (100.00)	38 (97.44)	1
CYC	52 (85.25)	19 (86.36)	33 (84.62)	1
MTX	1 (1.64)	1 (4.54)	0	0.34
MMF	1 (1.64)	0	1 (2.56)	1
Plasmapheresis	34 (55.74)	16 (72.73)	18 (46.15)	0.06
Rituximab	10 (16.39)	5 (22.73)	5 (12.82)	0.47
Death				<0.01 (total death)
Before 2000	7 (11.48)	0	7 (17.95)	0.55
After 2000	7 (11.48)	0	7 (17.95)	0.03

–*, not applicable*.

In this series, 41 patients had respiratory symptoms at the onset of their disorders (41/61); among them, patients who received ECMO treatment had a higher proportion (18/22 vs. 23/39), but without statistical significance. Except for our patients and the patient reported by Falk et al. (58), all patients in this series presented with symptoms of extrapulmonary involvement (59/61). Regarding respiratory failure, DAH was the main reason for GPA patients developing ARF (59/61), while in the other two cases, airway stenosis-related ventilation dysfunction was considered to be the etiology. There were three cases infected with cytomegalovirus, one with pneumocystis jirovecii pneumonia, and one with mucormycosis after immunosuppressive treatment.

By examining imaging manifestations, after excluding patients without radiological information, we found that the extent of involvement was related to the severity of the respiratory failure. Patients with DAH mainly displaying infiltration (45/58) and other imaging findings such as cavities (9/58), consolidation (13/58), and nodules (15/58) were relatively rare. Comparing patients who received and did not receive ECMO (Table 3), the infiltration proportion was much higher in those who received ECMO treatment (15/18 vs. 30/39), while the number of nodules was much lower (3/19 vs. 12/39). Among the two cases of airway stenosis, stenosis was detected in the radiological image directly in one patient, while another displayed pneumothorax and emphysema, which indirectly indicated airway stenosis. However, there were no statistical differences between the two groups in terms of radiological manifestations.

Except for cases not mentioned, tested, or classified, most of the patients in the series had a positive c-ANCA (42/46), and the most frequent antigen type was PR3 (35/38). P-ANCA (2/46) and MPO (2/38) were positive in only two patients, while c/p-ANCA double-positive and PR3/MPO double-positive were found in one patient each.

About ECMO, 22 patients received ECMO support in this series (22/61). Among those ECMO patients, 17 received VV-ECMO (20/22), one received VA-ECMO (1/22), and one received VA-ECMO and VV-ECMO (1/22) sequentially. All patients, except the one who died before diagnosis, were administered glucocorticoids (60/61); cyclophosphamide was another important immunosuppressive drug for combinational utilization (52/61), while other immunosuppressive drugs such as methotrexate (1/61) and mycophenolate mofetil (1/61) were seldom used. Ten patients in this series received rituximab, a drug developed in recent years. Additionally, plasma exchange was also widely used in this series (34/61), and the proportion of patients receiving ECMO was 16/22, while that in non-ECMO was 18/39 (Table 3).

Finally, 14 patients died in this series due to ARF (14/61); none of them received ECMO support (Table 3), and the results showed a higher survival rate of patients in the ECMO group.

## Discussion

GPA is a type of AAV; 85% of the patients with GPA show positive ANCA, mainly PR3-ANCA, and only a few demonstrate MPO-ANCAs (62). GPA is a multisystem vasculitis syndrome characterized by granulomatous lesions and necrotizing vasculitis, and all small blood vessels could be involved. The clinical symptoms of GPA vary and cover a wide range of symptoms, including fever, fatigue, weight loss, poor appetite, anorexia, arteritis, night sweats, cough, and dyspnea (63). Treatment strategies generally involve 3–6 months of remission induction plus more than 24 months of maintenance treatment using glucocorticoids in combination with immunosuppressors; sometimes, rituximab is added during the induction, and maintenance period (7).

More than 90% of patients with GPA have upper or lower respiratory tract involvement; however, the clinical manifestations can be asymptomatic to life-threatening respiratory failure. Among them, DAH and subglottic stenosis are the most common characteristics of GPA respiratory tract involvement (64, 65). Including the case, we reported that the main cause of respiratory failure was DAH in our series, and subglottic stenosis was relatively rare. It is noteworthy that patients with GPA may be associated with opportunistic infections of the lungs due to immunosuppressive therapy.

GPA-related DAH occurs in 7–45% of patients with GPA (65, 66). Fewer than 10% of cases with DAH are serious with life-threatening conditions (29); most of them are GPA patients with alveolar hemorrhage, and their mortality rate is six times that of those without alveolar hemorrhage (67). Among patients with GPA, 60% are caused by DAH (43). Any degree of DAH should be considered a serious manifestation of the disease as it can quickly progress to respiratory failure (62). The underlying pathophysiological mechanism of GPA-related DAH is linked to antibodies related to ANCA, which interact with inflammatory cells (neutrophils, monocytes, and lymphocytes) and cytokines (particularly Th1 cytokines, namely, tumor necrosis factor and interferon-γ) and then initiate and perpetuate vasculitis (68, 69). The basement membrane of capillaries is destroyed during these processes, and erythrocytes are allowed to enter the alveolar space with fluid (69). These processes damage the gas exchange of the affected lung area. However, the symptoms of DAH, including dyspnea, hypoxia, anemia, and lack of imaging findings of diffuse alveolar filling, are not specific; however, hemoptysis is relatively rare in DAH. In clinical practice, when a case with imaging findings indicates extensive infiltration but is refractory to anti-infective treatment, bronchial alveolar lavage under bronchoscopy is recommended to ascertain the occurrence of DAH (62), and immunological examination should also be conducted to obtain a definitive diagnosis that can help clinicians arrange immunosuppressive treatment earlier.

Moreover, similar to DAH, the symptoms of subglottic stenosis lack specificity, ranging from coughing and dyspnea to life-threatening stridor (65). Unlike DAH, subglottic stenosis generally does not show any positive findings on radiology, bronchoscopy is needed to confirm the diagnosis, and the severity of respiratory involvement is related to the location, length, and degree of stenosis (65). In addition, rapid progress to respiratory failure may occur among these patients soon after admission (41, 58).

Our patient has a severe case of the disease that progressed rapidly to respiratory failure within 3 days of admission, and respiratory support was upgraded from HFNC and NIPPV to intermittent mandatory ventilation (IMV) and ECMO in a short period. Additionally, the patient had no extrapulmonary manifestations, and severe infective pneumonia was the first clinical diagnosis. GPA was not diagnosed until a positive ANCA result was reported. At this time, ECMO was used for approximately 12 h. This case showed that GPA may present with unspecified clinical symptoms and radiological manifestations with rapid progress into respiratory failure, which leads to difficulties in early diagnosis and proper treatment.

Therefore, when respiratory symptoms occur in patients with GPA, etiological treatment and powerful respiratory support should be provided as soon as possible. Glucocorticoids and cyclophosphamide are the first-line drugs for impact and maintenance treatment. Rituximab and plasmapheresis should be considered when feasible (7). Respiratory support should be chosen based on oxygenation conditions, and HFNC should be upgraded early to IMV or ECMO when necessary and available (5) to further timely diagnose and treat. In this case series, all patients received ECMO as they were unable to maintain oxygenation through basic respiratory support; among them, one patient received VV-ECMO and VA-ECMO sequentially due to circulatory dysfunction related to mediastinal emphysema.

ARF is one of the indications for ECMO support. Since the first patient with ARDS was rescued using ECMO in 1972 (70), it has been increasingly used in ARF (71). Without ECMO support, our patient would not have survived. In our series, none of the patients could maintain oxygenation under mechanical ventilation before ECMO, indicating that ECMO can improve the prognosis of respiratory failure caused by GPA (59) and provide the possibility of later recovery. In other studies, such as the conventional ventilatory support vs. extracorporeal membrane oxygenation for severe acute respiratory failure in 2009, ECMO significantly improved the disability-free survival rate of patients with severe ARF (72). ECMO to rescue lung injury in severe ARDS (EOLIA) in 2008 showed that ECMO can significantly reduce the probability of 60-day treatment failure in patients with severe ARDS and increase the days of improved oxygenation, and no renal failure has occurred (73). However, whether ECMO can improve the prognosis of ARF remains controversial. There was no statistical significance in terms of prognosis between ECMO and non-ECMO users among patients with ARDS in the EOLIA study (73). Among patients with immune-related DAH, ECMO did not improve the survival rates (20 vs. 38%, *p* = 0.323) (74). Although these studies showed negative results, we should note that the early termination of the EOLIA study and the high proportion of patients who received remedial treatments from the traditional treatment group transferred to the ECMO group may lead to no statistically significant difference in mortality between the two groups. In studies of patients with ARDS owing to DAH (74), those who required ECMO generally had a lower oxygenation index (87 vs. 62, *p* = 0.017), more days of mechanical ventilation (21 vs. 9.5, *p* < 0.001), and higher alveolar–arterial gradient (450 vs. 586, *p* = 0.017), which indicates that even if these two groups of patients are included in the study under the same criteria, the oxygenation index may be much worse in patients who used ECMO, which may generate negative results.

Hence, regardless of the conclusions of future clinical studies on whether ECMO can improve the prognosis of patients with GAP-related respiratory failure, we recommend ECMO as a transitional method for those with respiratory failure caused by GPA who cannot maintain oxygenation through mechanical ventilation. Moreover, ECMO could win some time for these patients to be correctly diagnosed and treated later (74).

## Conclusion

ECMO provides the final respiratory support for GPA-related critical respiratory failure and assists patients who cannot maintain oxygenation using mechanical ventilation for further definitive diagnosis and treatment. However, whether it can improve the clinical outcomes of GPA-related respiratory failure remains to be further studied.

## Data Availability Statement

The original contributions presented in the study are included in the article/supplementary material, further inquiries can be directed to the corresponding author/s.

## Ethics Statement

Ethical review and approval was not required for the study on human participants in accordance with the local legislation and institutional requirements. The patients/participants provided their written informed consent to participate in this study.

## Author Contributions

RW and YL reviewed the lectures and wrote the manuscript. WenY, XM, WeiY, YZ, RL, and YF collected and arranged the materials. CH, QC, and PP viewed the complete manuscript. All authors contributed to the article and approved the submitted version.

## Conflict of Interest

The authors declare that the research was conducted in the absence of any commercial or financial relationships that could be construed as a potential conflict of interest.
